# An unusual case of gallstone ileus with rare spontaneous resolution and literature review

**DOI:** 10.1093/jscr/rjaf634

**Published:** 2025-08-15

**Authors:** Aram Almasaud, Lara Alkhelaiwy, Abdulmehsen Alzakari

**Affiliations:** General Surgery Department, Security Forces Hospital Program, Riyadh 11481, Saudi Arabia; General Surgery Department, Specialized Medical Center, Riyadh 11372, Saudi Arabia; General Surgery Department, King Abdulaziz Medical City, Ministry of National Guard-Health Affairs, Riyadh 11372, Saudi Arabia

**Keywords:** gallstone ileus, bowel obstruction, spontaneous resolution, conservative management, case report, elderly patient

## Abstract

Gallstone ileus is a rare cause of mechanical bowel obstruction, typically affecting elderly patients with a history of gallstones. It results from the migration of a gallstone through a cholecystoenteric fistula into the bowel, most commonly lodging in the ileum. We report a case of a 59-year-old female who presented with small bowel obstruction caused by a 22 mm gallstone. During diagnostic laparoscopy, no obstructing stone was found, and subsequent imaging revealed its migration to the descending colon. The patient was successfully managed conservatively without surgical intervention. This case emphasizes the importance of considering gallstone ileus in elderly patients presenting with obstruction and demonstrates that conservative management may be an option in select cases, particularly when the stone progresses distally. Repeat imaging and a multidisciplinary approach are essential for optimal outcomes.

## Introduction

Gallstone ileus is a rare but important cause of mechanical bowel obstruction, most frequently seen in elderly patients with a history of cholelithiasis. It occurs when a cholecystoenteric fistula—most commonly between the gallbladder and duodenum—allows one or more gallstones to enter the gastrointestinal tract. These stones can migrate and cause obstruction, most often in the terminal ileum, although more distal locations such as the colon may also be affected.

The diagnosis relies primarily on imaging, particularly computed tomography (CT), which can identify the ectopic gallstone, the fistulous tract, and the site of obstruction. The standard treatment is surgical removal of the stone via enterolithotomy. However, in rare cases where the gallstone passes spontaneously into the colon, conservative (non-operative) management may be a viable alternative. This approach is supported by select case reports, including the case presented here.

## Case report

We report the case of a 59-year-old female with no significant past medical or surgical history who presented to the emergency department with a 2-day history of abdominal pain. The pain initially started in the epigastric region, was intermittent, and later migrated to the right lower quadrant by the following morning. It gradually increased in severity and became continuous. The patient experienced three episodes of bilious vomiting and reported no bowel movement for 2 days, with minimal flatus passed on the morning of presentation. She denied fever, dark urine, pale stools, or similar episodes in the past.

On examination, vital signs were as follows: blood pressure 158/87 mmHg, heart rate 104 beats per minute, respiratory rate 23 breaths per minute, and temperature 36.6°C. The abdomen was mildly distended with tenderness in the epigastric region and right upper quadrant, but there were no signs of peritonitis such as rebound tenderness or a positive Murphy’s sign.

Laboratory investigations showed:

- Total bilirubin: 11.9 μmol/l
- Alkaline phosphatase: 212 U/l
- Alanine transaminase: 71 U/l
- White blood cell count: 10.7 × 10^9^/l
- Hemoglobin: 14 g/dl
- Lactate: 2.1 mmol/l

**Figure 1:**
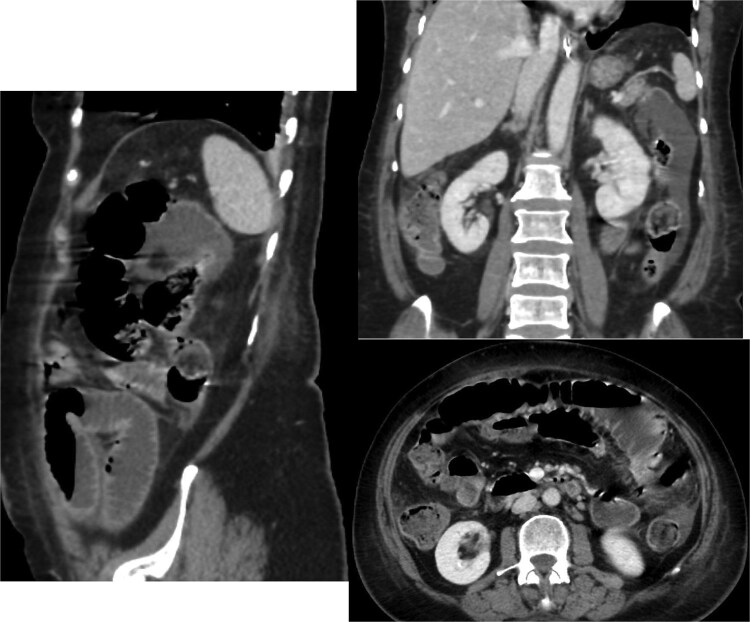
Contrast-enhanced CT scan of the abdomen and pelvis showing the small bowel loops are dilated reaching up to 35 mm. There is a transition zone with 22 mm gallstone in the distal ileum with distally collapsed ileal segments and collapsed large bowel loops representing high-grade obstruction. The gallbladder appears irregular with thickened wall and significant surrounding fat stranding. There is a fistula between the gallbladder and the second part of the duodenum.

A CT scan of the abdomen revealed (Fig. 1):

- A thickened gallbladder with a cholecystoduodenal fistula
- A 22-mm gallstone impacted in the distal ileum causing high-grade small bowel obstruction
- No signs of bowel ischemia, pneumoperitoneum, or intra-abdominal collections
- No evidence of diverticulitis

Based on these findings, the diagnosis of gallstone ileus secondary to a cholecystoenteric fistula was made.

### Hospital course

Patient was taken for diagnostic laparoscopy. Intraoperatively, no obstructing gallstone was found in the ileum, and there was no evidence of bowel ischemia or peritonitis. This prompted the surgical team to reconsider the management approach.

**Figure 2:**
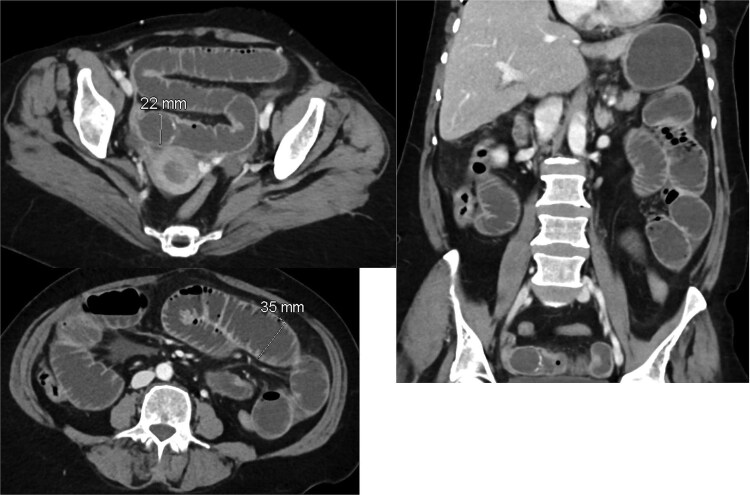
Interval passage of the previously small bowel obstructing gallstone seen now at the mid descending colon with proximal colonic distention however no signs of mechanical bowel obstruction. The small bowel loop is dilated and fluid-filled without definite transition zone; there is associated mild small bowel thickening, findings are likely representing postoperative changes.

Given the absence of a stone in the ileum, we continued with conservative management. A repeat CT scan was performed 2 days later (Fig. 2). The new imaging demonstrated that the gallstone had passed through the ileum and had reached the mid descending colon. As the stone had migrated to the colon, and the CT showing picture of paralytic ileus, the decision was made to continue with conservative management, including monitoring the patient’s symptoms and providing supportive care.

Over the next several days, the patient’s symptoms progressively improved and was passing flatus and minimal amount of loose stool on Day 3 post-op and had one episode of bilious vomiting, minimal quantity. She was discharged on Day 5 post-op in stable condition after demonstrating full recovery, with instructions to follow-up with her primary care provider.

## Discussion

This case highlights the importance of including gallstone ileus in the differential diagnosis of elderly patients presenting with signs of bowel obstruction, particularly in those with a known history of cholelithiasis. Although gallstone ileus is a relatively rare complication, it is associated with significant morbidity and mortality if not promptly diagnosed and managed [1]. The condition usually involves the impaction of a gallstone within the small intestine, most commonly at the ileocecal valve, and typically requires surgical intervention for stone removal. In some cases, the associated cholecystoenteric fistula may also need to be addressed surgically [2].

In this case, despite the gallstone measuring approximately 22 mm—well within the size range known to cause obstruction [3]—the patient experienced spontaneous resolution due to the stone’s migration into the colon. This is a rare event, as the ileocecal valve generally represents a mechanical barrier that prevents the passage of larger stones. The mechanism behind such spontaneous passage remains speculative but may involve intermittent obstruction, favorable stone morphology, or changes in bowel motility.

The initial presentation, consistent with high-grade small bowel obstruction, led to diagnostic laparoscopy in accordance with the standard management for gallstone ileus. However, the absence of the obstructing stone intraoperatively, and its subsequent identification in the descending colon on repeat imaging, underscores the dynamic nature of this condition. This case supports the utility of serial imaging in managing suspected gallstone ileus, particularly when operative findings are inconclusive [4–8] (Table 1).

**Table 1:** Published cases of small bowel obstruction due to gallstone ileus that had successfully been managed conservatively [4–7]

	**Age**	**Gender**	**Symptoms and duration**	**Stone size**	**Location**	**Time to resolution of symptoms**
Tandon A, 2013 [4]	60 years old	Female	Vomiting, pain, distention, and constipation for 3 days	40 mm	Ilecocecal junction	3 days
Takata H, 2015 [5]	85 years old	Female	Abdominal pain and vomiting for 1 day	28 mm	Small bowel	14 days
Takahashi K, 2018 [6]	65 years old	Male	Abdominal pain and vomiting, duration not mentioned	32 mm	Jejunum	4 days
Brogna B, 2022 [7]	84 years old	Female	Left abdominal pain and coffee-ground vomitus, sudden in 1 day	26 mm	Jejunum	7 days
Our case, 2015	59 years old	Female	Abdominal distention, nausea, and vomiting, not passing bowel motion for 2 days	22 mm	Distal ileum	3 days

Although surgery remains the mainstay of treatment for gallstone ileus [9], this case illustrates that conservative management may be a viable alternative in highly select cases where the stone has migrated beyond the ileum and the patient remains clinically stable. Nonetheless, it is important to emphasize that spontaneous resolution is an exception rather than the rule, and close monitoring is essential to detect any signs of ongoing or recurrent obstruction.

This case also highlights the importance of early diagnosis using appropriate imaging techniques—especially CT scans, which can detect the gallstone, the site of obstruction, and the fistulous connection. Furthermore, a multidisciplinary approach involving surgeons, radiologists, and gastroenterologists is essential to provide optimal care in such complex cases.

## Conclusion

This case illustrates a rare spontaneous resolution of gallstone ileus and reinforces the importance of considering it in elderly patients with obstructive symptoms. Although surgery is the primary treatment, conservative management may be suitable in selected scenarios, especially if imaging confirms stone migration without ongoing obstruction. This case highlights the critical role of imaging and clinical monitoring in guiding treatment decisions.
